# Improving operational preparedness for armored vehicle casualty rescue: effectiveness of simulation-based training with a novel extrication device

**DOI:** 10.3389/fpubh.2026.1910210

**Published:** 2026-07-27

**Authors:** Antonio Javier Segura-Fornieles, Verónica V. Márquez-Hernández, Alba García-Viola, Mª Carmen Rodríguez-García, José Miguel Garrido-Molina

**Affiliations:** 1 Regimiento Acorazado "Alcázar de Toledo" n° 61, Brigada Guadarrama XII, Madrid, Spain; 2 Department of Nursing, Physiotherapy and Medicine, Faculty of Health Sciences, Research Group for Epidemiology and Public Health CTS-1127, University of Almeria, Almeria, Spain; 3 Distrito Sanitario Almería, Almeria, Spain; 4 Centro de Emergencias Sanitarias 061, Servicio Andaluz de Salud, Almería, Spain

**Keywords:** military emergency, patient extrication, prehospital trauma care, search and rescue, tactical rescue

## Abstract

**Background:**

In recent years, the medical training of military personnel has significantly increased with the aim of providing tactical care in the field to save or stabilize lives until specialized healthcare personnel can begin treatment.

**Objectives:**

This study aimed to assess the effectiveness of a training intervention using the Snaid® extrication device on infantry personnel of the Army, whose primary weapon system is the CC Leo 2E.

**Methods:**

A quasi-experimental one-group pretest-posttest study was conducted. A total of 156 military personnel participated in the study. Sociodemographic data such as sex, age, and prior training on the use of the device were collected. Additionally, measures such as knowledge, observation of the intervention, skill, and confidence were assessed. Data analysis was performed using SPSS statistical software, version 29.

**Results:**

The results showed a higher level of knowledge in the post-intervention phase. Additionally, statistically significant differences were found in all observed parameters after the intervention. The average response time also decreased, and statistically significant differences were observed in skill and confidence in the post-intervention phase.

**Conclusion:**

The findings indicate significant improvements in knowledge, reduction of extrication time, ability to teach others, and the participants’ preparedness to apply the device after the intervention.

## 1 Introduction

Road traffic accidents are a major global public health problem, with a significant mortality, morbidity and socio-economic costs (1, 2). The World Health Organization (WHO) estimates 1.19 million deaths and 20–50 million injuries annually worldwide, making road traffics accidents a leading cause of death among young people (3). In Spain, the 2023 Road Accident Report recorded 1,806 fatalities across 101,306 collisions, with 9,265 hospitalized and 124,266 non-hospitalized casualties (4). Heavy vehicles, were involved in 7.7% of accidents occurring during working hours, underscoring vehicle mass as a key severity factor (5). Globally, road traffic accidents are estimated to cost around 3% of countries’ gross domestic product, reflecting their enormous health and economic impact (6).

Evidence generated in civilian settings is highly relevant to operational military environments (7). The injury mechanisms of high-energy collisions—sudden deceleration, kinetic energy transfer, and entrapment—are comparable between conventional road accidents and incidents involving heavy reinforced vehicles, providing a shared biomechanical framework for blunt trauma (8, 9). In armored vehicles, however, these mechanisms are compounded by cabin confinement, limited ergonomics, bulky tactical equipment, and hostile environments (8–10), which increase injury severity and complicate extrication and initial medical care (11–13).

In 2023, the Spanish Ministry of Defense reported 2 fatalities among 22 land-vehicle accidents in the Armed Forces, three of which involved armored vehicles colliding with obstacles (14). The Leopard 2E battle tank (CC Leo 2E) weighs 62 tons, reaches 70 km/h (43.5 mph) and is operated by a four-person crew (15, 16) armored warfare with such vehicles remains predominant in modern conflict (17) and injured combatants typically sustain limb and craniofacial trauma (18).

Prehospital trauma care is the essential pillar supporting the entire rescue chain: recognizing life-threatening injuries enables proper triage and timely treatment (19–21) and spinal motion restriction is standard practice for polytraumatized patients (22). Injury patterns vary by mechanism – extremities, head, and neck are most commonly affected in traffic accidents (23) while in the military field, musculoskeletal injuries are the leading cause of medical discharge and reduced deployment capability (24, 25).

Extrication, the process of treating and preparing a casualty for release from a vehicle (26), involves complete spinal motion restriction, which takes a significant amount of time, delays surgical intervention, and is not supported by high-quality evidence (27). In response to this challenge, new casualty extrication devices have been introduced. Among them, Snaid® has been described by its manufacturer as a rescue tool designed to provide cervical motion restriction, reduce the risk of secondary spinal injury, and enhance the efficiency of extrication operations (28).

Military healthcare training has expanded significantly in recent years (29–31) to provide tactical field care until specialized personnel can begin treatment (32). Despite advances in healthcare training for military personnel, only two studies have identified the use of the aforementioned rescue device (33, 34). Pons-Claramonte et al. (33) evaluated the biomechanics of the spine during a simulated vehicle extrication, comparing spinal alignment with the time required for rescue. On the other hand, Márquez-Hernández et al. (34) focused their study on training for this device, aimed at future healthcare professionals. Neither assessed device performance in confined, real-world operational settings or during coordinated team extractions—a critical gap given that multi-team coordination and shortened extrication times are decisive for casualty survival, particularly as simulation-based training becomes more central to military medical education.

Addressing this is particularly relevant given the importance of rapid, safe casualty extraction in military trauma care. We hypothesized that simulation-based training in the use of the Snaid® rapid extrication device would significantly improve military healthcare personnel’s knowledge, technical performance, and self-reported readiness to use the device in armored vehicle casualty extrication. Therefore, the aim of this study was to evaluate the effectiveness of a structured simulation-based training intervention using the Snaid® extrication device among infantry personnel operating the Leopard 2E main battle tank. Specifically, the study examined changes in knowledge, self-efficacy, procedural performance, confidence, technical skills, and extrication time following training.

## 2 Methods

### 2.1 Design

A quasi-experimental one-group pretest-posttest design was used.

### 2.2 Participants

A total of 156 military personnel participated in the study. Participants were recruited using a convenience sampling strategy. The inclusion criteria were: (a) active military personnel assigned to the 61st Tank Infantry Battalion of the XII Brigade of the Spanish Army, and (b) age between 18 and 50 years. The exclusion criterion was having less than 1 year of active military service.

An *a priori* sample size calculation was performed for the primary pre–post analysis (paired-samples t-test, two-tailed *α* = 0.05, power = 80%). Based on previous studies evaluating simulation-based and emergency training interventions, a large effect size (Cohen’s d = 0.80) was assumed together with a moderate correlation between paired measurements (*r* = 0.50). Under these assumptions, the minimum required sample size was 15 participants. The final sample (*N* = 156) substantially exceeded this requirement.

To further assess the adequacy of the achieved sample, a *post-hoc* sensitivity analysis was conducted. With *N* = 156, *α* = 0.05, and 80% statistical power, the minimum detectable effect size was Cohen’s d = 0.23, indicating that the study was adequately powered to detect even small-to-moderate intervention effects.

### 2.3 Measurements

Sociodemographic data such as sex, age, and prior training on the use of the device were initially collected. Additionally, measures such as self-efficacy, knowledge, intervention observation, skill, and confidence were assessed. Each of these measures is described below.

Self-efficacy: A question was used with a response range from 0 (not capable) to 10 (fully capable). The higher the score, the higher the level of self-efficacy (24).

Knowledge: To measure the level of knowledge, the same tool used in the study by Márquez et al. (24) was employed. It is a questionnaire consisting of 10 questions with 4 response options, where only one answer was correct. The score range was from 0 to 10, with higher scores indicating greater knowledge. The Cronbach’s alpha in the original study was 0.802. In this research, the Cronbach’s alpha was 0.863.

**Figure 1:**
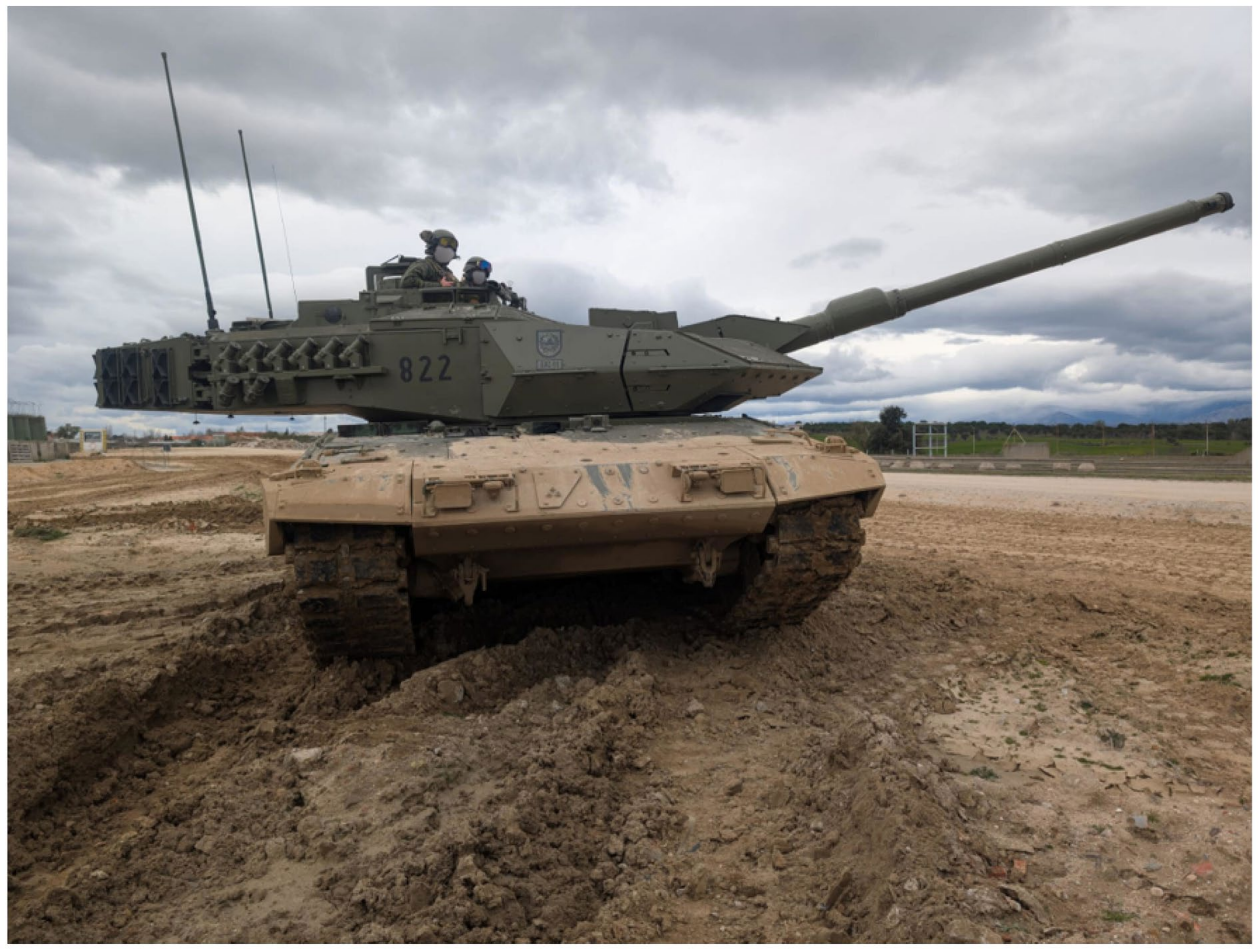
Turret at 9.

Observation of the intervention: To measure the observation of the intervention, the following pre- and post-training variables were collected according to the manufacturer’s recommendations:

Position of the vehicle turret: A reference system similar to the position of clock hands was used: Turret at 12: The turret of the CC Leo 2E is oriented directly forward, aligned with the direction of movement of the hull. Turret at 9: The turret is oriented to the left (Figure 1).

Variables assessed included: position of the casualty; perform XABCDE before placing the device; proper performance of XABCDE; placement of the device with the central mark aligned with the chin; cross the device at the level of the neck, moving both ends forward; ensure the device is properly adjusted to the casualty’s neck; slide both ends under the armpits so they protrude from the back; place the upper limb immobilizer; check whether the immobilizer is properly/incorrectly placed; place both slings to increase the length of the device; ensure the casualty’s feet are free; effectively coordinate the extrication with the team pulling the slings; prevent trauma inside the CC Leo 2E during extraction; perform XABCDE after extrication; and extrication time.

Skill and Confidence: To measure skill and confidence, the following questions were used, based on previous studies: (24). On a scale from 0 to 10, indicate your confidence in using the device; on a scale from 0 to 10, indicate your skill in using the device; comfort in using the device; on a scale from 0 to 10, indicate to what extent you would recommend the use of the device; do you feel capable of teaching others how to use the device?; do you feel prepared to use the device?

### 2.4 Data collection

**Figure 2:**
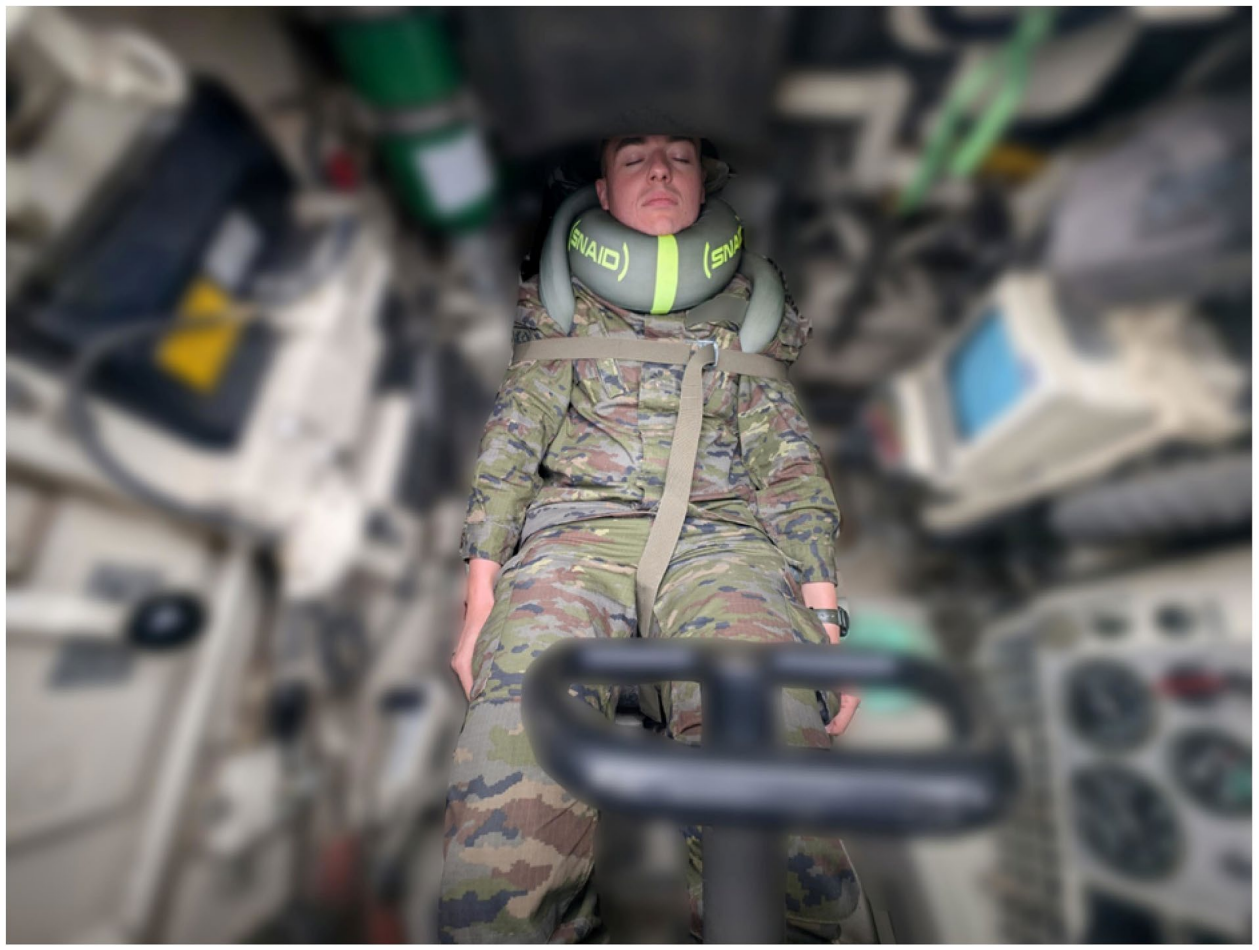
Placement of the Snaid® device on the driver of the CC Leo 2E.

The study was conducted at the “El Goloso” Military Base during February 2025. First, the participants were informed about the study procedure, its objectives, the voluntary nature of their participation, confidentiality, and the possibility of withdrawing from the study at any time. After completing the participant information sheet and the informed consent, the intervention began, which was divided into 3 phases:

Phase 1: Completion of the self-efficacy and knowledge scales before the training session. Once completed, the participants were divided into two groups, which were further subdivided into groups of 4, following the actual crew configuration of the battle tanks. A monitoring of the device placement within the CC Leo 2E was conducted in two different turret positions (turret at 12 and turret at 9), performing the extrication of the tactical positions of the driver (Figure 2) and the loader. Turret position and tactical role were assigned according to real operational crew configurations rather than through randomization. During this monitoring, the lead rescuer was informed that the hypothetical casualty was unconscious, breathing, with the upper right limb completely bloodied in bright red. The correct placement and extrication time were evaluated. Extrication time was measured using a standard handheld stopwatch operated by a trained member of the research team. All measurements were performed by the same observer across all simulations to ensure consistency and standardization of data collection. Due to the nature of the simulation setting, blinding of the assessor was not feasible, and inter-rater reliability was not assessed. The observer responsible for data collection was a military nurse with experience in tactical casualty care and simulation-based training, who had been previously trained in the study protocol and assessment procedures.

Hemorrhage control was expected as part of appropriate XABCDE management; however, it was not recorded as an independent outcome variable. Instead, correct application of XABCDE was assessed using a procedural checklist. The duration of the XABCDE sequence was not separately measured or scored, as the study focused on overall procedural efficiency.

Phase 2: A two-hour theoretical-practical training session was conducted, which included an introduction to the device, the potential benefits of applying the device in the CC Leo 2E, a video demonstrating proper placement, two videos illustrating when not to use the device, and a healthcare training on XABCDE assessment. The training was conducted by a member of the research team who is a qualified military nurse. To ensure standardization, a structured training script and protocol were developed and previously reviewed and agreed upon by all members of the research team. At the end of the theoretical phase, each participant practiced the placement of the device, following the manufacturer’s recommendations and steps. During this practical training, participants received individualized performance feedback.

**Figure 3:**
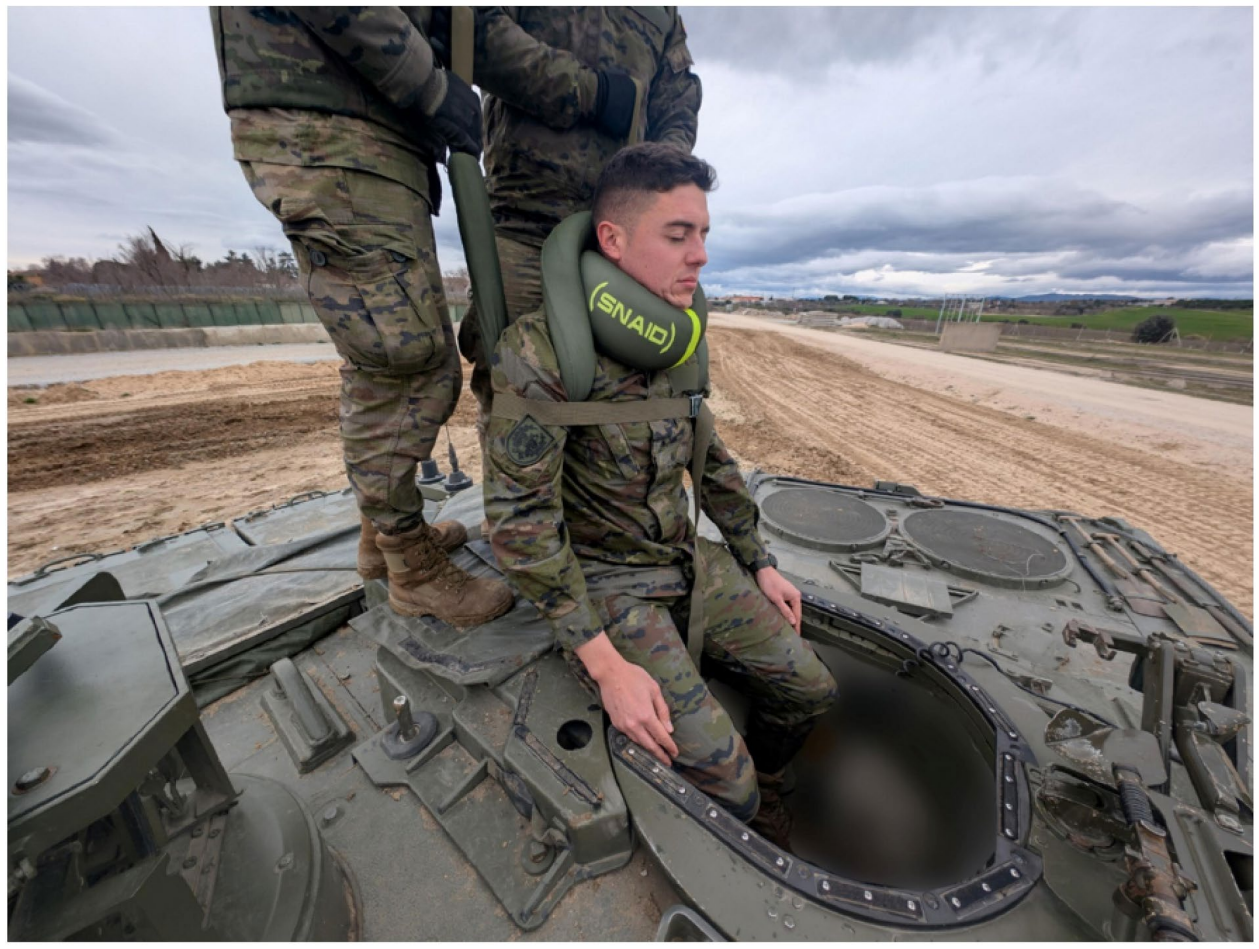
Completion of extrication from the CC Leo 2E via the top hatch.

Phase 3: Once the theoretical-practical session was completed and the participants’ questions were addressed, the use of the extrication device in the vehicle was reassessed in the same groups as in Phase 1. The personnel extricated the simulated casualty from the same turret position and the same tactical position, with the same symptoms described as at the beginning. The extrication time was measured from the moment the device touched the casualty’s neck until it was extracted through the top hatch of the CC Leo 2E (Figure 3). After the device monitoring, the participants completed the self-efficacy and knowledge survey again.

### 2.5 Ethical aspects

The study was approved by the Research Ethics Committee for Medicinal Products of the Central Defense Hospital (Code 20/24). All participants signed the informed consent document and a participant information sheet, being previously informed about the study’s objective, its characteristics, as well as the anonymous treatment and confidentiality of the data. Additionally, the guidelines established by international protocols and the Helsinki Declaration were followed.

### 2.6 Data analysis

Data analysis was performed using the SPSS statistical software, version 29. Descriptive analysis of the data was conducted. For quantitative variables, mean and standard deviation were analyzed, while for categorical variables, frequencies and percentages were calculated. The Chi-square test and Fisher’s exact test were used. After checking the distribution of variables using the Kolmogorov–Smirnov test, the non-parametric Mann–Whitney U test and Wilcoxon test were applied. Spearman’s correlation test was also employed. A *p*-value of <0.05 was considered statistically significant.

## 3 Results

### 3.1 Sociodemographic characteristics of the participants

A total of 156 participants took part in the study, of which 94.9% (*n* = 148) were men and 5.1% (*n* = 8) were women. The average age of the participants was 26.46 years (SD = 6.41), with an age range between 18 and 49 years. Regarding prior experience, 60.3% (*n* = 94) of the participants had no experience in using the device, whereas 39.7% (*n* = 62) had previously used it during training.

### 3.2 Self-efficacy and knowledge

The pretest self-efficacy score was 5.76 (SD = 2.58), while the post-intervention self-efficacy score was 7.72 (SD = 1.91), showing statistically significant differences between the two measurements (*p* < 0.001). No statistically significant differences were found when comparing self-efficacy scores with sex (*p* = 0.782) or age (rs = 0.001; *p* = 0.988).

Regarding knowledge, statistically significant differences were found when comparing the pre-test and post-test results (*p* = 0.004). Specifically, participants had an average score of 5.66 (SD = 1.15) in the pre-test and an average score of 8.82 (SD = 1.17) in the post-test.

### 3.3 Intervention

In the intervention, 53.8% (*n* = 84) of the extrications were performed in position 12, while 46.2% (*n* = 72) were performed in position 9. Regarding the position of the casualty, 53.8% (*n* = 84) were positioned as the driver, and 46.2% (*n* = 72) were positioned as the loader. When comparing the pre- and post-intervention measurements, statistically significant differences were found across all parameters (Table 1).

**Table 1:** Comparison of pre-post intervention results.

Item	Pretest		Posttest		*p*
	Yes	No	Yes	No	
Perform XABCDE before placing the device	53 (34%)	103 (66%)	156 (100%)	-	<0.001
Place the device aligning the central mark with the chin	89(57.1%)	67 (42.9%)	153(98.1%)	3(1.9%)	<0.001
Cross the device at the level of the neck, moving both ends forward	82(52.6%)	74(47.4%)	156(100%)	-	<0.001
Ensure that the device is properly fitted to the casualty’s neck	47(30.1%)	109(69.9%)	139(89.1%)	17(10.9%)	<0.001
Slide both ends under the armpits so that they protrude from the back	76(48.7%)	80(51.3%)	156(100%)	-	<0.001
Place the upper limb immobilizer	60(38.5%)	96(61.5%)	145(92.9%)	11(7.1%)	<0.001
Place both slings to increase the length of the device	118(75.6%)	38(24.4%)	143(91.7%)	13(8.3%)	<0.001
Effectively coordinate the extraction with the team members pulling from the slings	84(53.8%)	72(46.2%)	143(91.7%)	13(8.3%)	<0.001
Prevents trauma inside the CC Leo 2E during the extraction	73(46.8%)	83(53.2%)	128(82.1%)	28(17.9%)	<0.001
Performs XABCDE after extrication	35(22.4%)	121(77.6%)	156(100%)	-	<0.001

Regarding the extrication time, the mean total intervention time was 3.23 min (SD = 1.18). Statistically significant differences were found between the pre- and post-intervention times (*p* < 0.001). Specifically, the average time in the pre-test was 3.41 min (SD = 1.28), while in the post-test it was 3.06 min (SD = 1.06).

Considering the position of the turret, statistically significant differences were found in the items detailed in Table 2 at the post-test moment. However, no statistically significant differences were found concerning sex or age.

**Table 2:** Results based on the turret position.

Item		Turret 9	Turret 12	*p*
Ensure that the device is properly adjusted to the casualty’s neck	Yes No	58(80.6%) 14(19.4%)	81(96.4%) 3 (3.6%)	0.002
Place the upper limb immobilizer	Yes No	61(84.7%) 11 (15.3%)	84(100%) -	<0.001
Place both slings to increase the length of the device	Yes No	59(81.9%) 13(18.1%)	84(100%) -	<0.001

### 3.4 Skill and confidence

The mean skill score in the pre-test was 5.74 (SD = 2.19), while in the post-test it was 8.67 (SD = 1.18), with statistically significant differences found (*p* < 0.001). Regarding confidence, the mean score in the pre-intervention was 7.08 (SD = 2.16), and in the post-intervention it was 8.72 (SD = 0.93), with statistically significant differences (*p* < 0.001). As for comfort, the pre-test score was 5.91 (SD = 1.59), and the post-test score was 7.58 (SD = 1.68), with statistically significant differences (*p* < 0.001). Finally, statistically significant differences were found when comparing the recommendation of device use (*p* < 0.001) between the pre-intervention moment (M = 7.87, SD = 1.71) and post-intervention (M = 9.05, SD = 0.83). The remaining variables are detailed in Table 3.

**Table 3:** Results on the ability to teach others how to use the device and preparation to use the device according to the measurement time.

Item	Pretest		Posttest		*p*
	Yes	No	Yes	No	
Ability to teach others how to use the device	51 (32.7%)	105(67.3%)	151(96.8%)	5(3.5%)	<0.001
Readiness to use the device	68 (43.6%)	88 (56.4%)	156 (100%)	-	<0.001

According to the turret position, statistically significant differences were found in confidence (U = 2144.500; Z = −3.347; *p* < 0.001), skill (U = 1591.000; Z = −5.367; *p* < 0.001), and comfort (U = 2398.000; Z = −2.264; *p* = 0.024) at the post-intervention stage. Specifically, the results were as follows: for confidence, turret position 9 (M = 9.02; SD = 0.78) and turret position 12 (M = 8.46; SD = 0.97). For skill, turret position 9 (M = 9.23; SD = 0.79) and turret position 12 (M = 8.19; SD = 1.24). For comfort, turret position 9 (M = 7.27; SD = 1.80) and turret position 12 (M = 7.84; SD = 1.53). No statistically significant results were found for the remaining variables.

## 4 Discussion

This study evaluated the effectiveness of a structured simulation-based training intervention using the Snaid® extrication device in a military population operating in confined armored-vehicle environments. The findings demonstrated significant improvements in knowledge, self-efficacy, technical skills, confidence, procedural performance, and extrication efficiency following the intervention. These results suggest that simulation-based training may be an effective strategy for enhancing preparedness and operational performance during casualty extraction in military settings.

Beyond the statistical improvements observed, these findings carry practical significance for military medical education. Reliable extrication and casualty-handling skills must be sustained under the cognitive and physiological stress typical of combat environments, conditions that are difficult to fully replicate in a training setting (33, 34). Recent work using immersive virtual reality to assess decision-making in tactical combat casualty care and mass-casualty scenarios among Spanish military personnel has similarly emphasized the value of realistic, high-fidelity simulation for evaluating performance under operational pressure, while noting that physiological stress markers and decision accuracy can vary considerably across individuals and scenario types (35). Incorporating structured, device-specific extrication training such as the one evaluated here into standard military medical education curricula could help institutionalize these skills ahead of deployment, complementing broader tactical combat casualty care programs.

The observed increase in knowledge and self-efficacy is consistent with previous research highlighting the educational benefits of simulation-based learning in emergency and trauma care. Simulation facilitates experiential learning through repetitive practice, immediate feedback, and exposure to realistic scenarios, all of which contribute to improved competence and confidence. In military environments, where decision-making often occurs under time pressure and adverse conditions, these educational gains may have important operational implications (33, 34).

Multiple studies have validated tools for assessing the correct application of medical equipment in simulation studies (36, 37). Similarly, in the study by Márquez Hernández et al. (34), the comparison of pre- and post-intervention scores in knowledge and self-efficacy of the device showed notable improvements in both aspects. This increase suggests not only an improvement in the participants’ technical skills but also enhanced confidence in the effective use of the extrication device.

The improvement in self-efficacy is a crucial aspect, as greater confidence in one’s abilities can translate into more precise execution of tasks in high-pressure and stressful situations (38). The improvement in knowledge ensures that participants are not only capable of performing the technique correctly (39), but also understand the context and the importance of each step to avoid exacerbating the injuries of the casualty.

The absence of significant associations between self-efficacy, skill, or confidence gains and either sex or age suggests that the training intervention produced consistent benefits regardless of participants’ demographic profile. This is consistent with a recent Spanish study of high-fidelity prehospital simulation among final-year medical students, which similarly found no significant differences in simulation performance by sex, age, or prior simulation training (40). Nonetheless, the marked sex imbalance in our sample (94.9% men), reflecting the composition of an armored infantry battalion, limits our ability to draw firm conclusions about sex-based differences in this specific population and warrants replication in more demographically balanced military cohorts. Similarly, although 39.7% of participants reported prior experience using the device, this study did not stratify outcomes by baseline familiarity; prior real-world or training experience has been identified elsewhere as a significant predictor of skill retention in simulation-based education, particularly for procedural competencies (41), and future analyses stratifying performance by prior device familiarity could help determine whether training gains are similarly sustained among novice and previously exposed personnel.

Extrication time is widely recognized as a critical determinant in emergency and prehospital care (27). A key consideration, however, is whether the observed reduction in response time (≈0.35 min) confers meaningful clinical or operational benefits beyond its statistical significance. Although the absolute difference may appear modest, evidence from studies indicates that even small decreases in time-to-intervention can yield measurable improvements in patient outcomes, particularly in time-sensitive conditions such as severe trauma, hemorrhagic shock or cardiac arrest (42–44). In these scenarios, short temporal intervals have been shown to exert a disproportionate influence on survival probability, neurological prognosis and overall clinical trajectory (45). Thus, while the reduction observed in this study is quantitatively limited, its potential operational relevance should not be underestimated within the context of high-risk, resource-constrained rescue environments.

The findings also showed significant improvements in the participants’ ability to teach others how to use the device and in their overall preparedness to use it after the training intervention. The significant change in their ability to teach others suggests that the intervention not only improved the participants’ individual skills but also provided them with the confidence and knowledge necessary to pass these skills on to others. The ability to teach is an essential component to ensure that the skills learned are disseminated and maintained within the team (46).

In addition to overall procedural performance, the marked increase in participants’ willingness to recommend the device (from a mean of 7.87 to 9.05) suggests high acceptability and perceived operational value among end users, a factor that may facilitate future adoption of the device within military units. Likewise, the significant improvements observed across nearly all individual checklist items evaluated pre- and post-intervention, including device alignment, sling placement, and coordination of the extrication maneuver, indicate that the training benefit extended beyond overall timing to the specific technical steps most likely to affect casualty safety during extraction.

Regarding the turret position, the 9 o’clock position showed better results in terms of skill and confidence; however, the 12 o’clock position may provide a slightly more comfortable working environment for the participants. It is possible that the 12 o’clock position allows for a more comfortable body posture during the extrication. Despite the lack of specific studies analyzing the position of a casualty inside an armored vehicle, the extrication principles share many similarities with those in the civilian sector. Key factors such as safety, available space, accessibility, environmental conditions, and the patient’s hemodynamic status determine the extrication techniques applied (47). These variables directly influence the choice of the safest and most efficient method to ensure the stabilization and rescue of the casualty.

The results obtained in this study suggest that the training intervention using the Snaid® extrication device could be applied to other operational contexts beyond the strictly military sphere. In particular, its potential application in civilian emergency settings, such as search and rescue teams, out-of-hospital emergency services or specialized units, is particularly relevant. The literature has demonstrated that simulation-based structured training significantly improves technical performance, decision-making, and coordination in highly complex scenarios, regardless of the environment in which it is applied (48, 49).

In this regard, the potential implementation of the device and the training program in water rescue operations constitutes an emerging line of research of great interest (50). Furthermore, the transfer of such interventions to military units in other countries or to local and regional teams could promote the standardization of procedures and improve interoperability between different response units. This is particularly relevant in contexts of international cooperation or major emergencies, where coordination between different teams is essential.

Therefore, future research should focus on evaluating the effectiveness of the Snaid® extrication device in diverse scenarios, including water rescues, complex urban environments and mass casualty situations, as well as analyzing its impact on clinical, operational and safety variables. This approach would help consolidate the available scientific evidence and broaden the scope of application of the device, contributing to the continuous improvement of rescue procedures and casualty care in critical situations (51).

The results of this study should be considered in light of several limitations. First, the study was conducted with a specific group of participants from a Tank Infantry Battalion, which may limit the generalizability of the results to other military units that do not work in confined spaces or to non-military personnel. Additionally, the use of a convenience sampling strategy may introduce selection bias; the voluntary participants in the study may have had a particular interest or motivation to improve their skills, and more motivated participants may have demonstrated greater improvements. The interventions and assessments were carried out in a controlled environment, which may not fully reflect the stressful and variable conditions of a real accident scenario. Furthermore, self-efficacy was assessed using a single-item question scored on a 0–10 scale rather than a validated multidimensional instrument. Therefore, this measure may not fully capture the different dimensions of the construct, and the observed changes in self-efficacy should be interpreted with caution. Moreover, the observed pre–post improvements in self-efficacy and knowledge should be interpreted cautiously, as they may be influenced by testing and repetition effects. This design is susceptible to bias, including learning effects, Hawthorne effect, and maturation, meaning that the observed improvements cannot be definitively attributed to the intervention alone. Furthermore, the long-term impact of the training was not assessed, leaving open the question of the sustainability of the observed benefits. Also, due to the novelty of the extraction device used, there are very few previous studies that provide a broad and solid comparative framework. Finally, although previous experience with the device was recorded, the analyses did not stratify participants according to prior experience. Consequently, it cannot be excluded that previous familiarity with the device may have influenced baseline performance or the magnitude of the improvements observed after the training intervention. We recommend continuing research on the device to validate our initial findings and expand knowledge about its effectiveness in real-life practice. Future studies should also incorporate improved methodological approaches, including multivariable analyses and controlled study designs, and validated multi-item scales for self-efficacy, skill, and confidence in military emergency training contexts.

## 5 Conclusion

This study demonstrated that a structured simulation-based training intervention using the Snaid® extrication device was associated with significant improvements in knowledge, self-efficacy, confidence, technical skills, procedural performance, and extrication efficiency among military personnel operating in confined armored-vehicle environments. These findings support the potential value of simulation-based extrication training as a component of military preparedness programs.

However, given the quasi-experimental design and the absence of a control group, causal inferences should be made with caution. Future studies using randomized controlled designs, long-term follow-up assessments, and objective clinical outcome measures are needed to confirm these findings and evaluate the applicability of the device across other military and civilian rescue contexts.

## Data Availability

The raw data supporting the conclusions of this article will be made available by the authors, without undue reservation.
